# The CagA toxin of *Helicobacter pylori*: abundant production but relatively low amount translocated

**DOI:** 10.1038/srep23227

**Published:** 2016-03-17

**Authors:** Luisa F. Jiménez-Soto, Rainer Haas

**Affiliations:** 1 Max von Pettenkofer-Institut für Hygiene und Medizinische Mikrobiologie, Ludwig-Maximilians-Universität, Pettenkoferstraße 9a, D-80336 München, Germany; 2 German Center for Infection Research (DZIF), LMU Munich, Germany

## Abstract

CagA is one of the most studied pathogenicity factors of the bacterial pathogen *Helicobacter pylori*. It is injected into host cells via the *H. pylori cag*-Type IV secretion system. Due to its association with gastric cancer, CagA is classified as oncogenic protein. At the same time CagA represents the 4^th^ most abundant protein produced by *H. pylori*, suggesting that high amounts of toxin are required to cause the physiological changes or damage observed in cells. We were able to quantify the injection of CagA into gastric AGS epithelial cells *in vitro* by the adaptation of a novel protease-based approach to remove the tightly adherent extracellular bacteria. After one hour of infection only 1.5% of the total CagA available was injected by the adherent bacteria, whereas after 3 hours 7.5% was found within the host cell. Thus, our data show that only a surprisingly small amount of the CagA available in the infection is finally injected under *in vitro* infection conditions.

Bacteria have developed several mechanisms to secrete proteins or to inject toxins into target cells. *Helicobacter pylori*, a bacterial pathogen associated with gastric pathologies, uses the *cag* Type IV secretion system (*cag*-T4SS) to inject the cytotoxin-associated antigen A (CagA) into host cells^1^. Once inside the cell, CagA is phosphorylated by the cellular Src and Abl kinases in the Glu-Pro-Ile-Tyr-Ala (EPIYA) motifs found on the C-terminal half of the protein^2^,^3^. Injected phosphorylated CagA is targeted to the apical junctional complex of the epithelial cell monolayer through the N terminus of the molecule^4^. Notably, CagA is sufficient to disrupt the mechanisms that maintain normal epithelial differentiation, including cell adhesion, cell polarity, and the inhibition of cell migration^5^. Via its phosphorylated EPIYA motifs CagA interacts with a large number of host cell proteins carrying SH2 domains^6^, which results in cytoskeleton rearrangements and in deregulation of several signal transduction pathways. All these effects appear to be controlled by different domains in the CagA protein, some of which are phosphorylation-dependent, especially those interfering with signaling cascades, whereas those which relate to the apical junctions are phosphorylation-independent^4^.

In order to achieve an efficient injection of CagA into the cell, *H. pylori* requires the Integrin Beta 1 (ITGB1) as receptor for the T4SS and a tight interaction with the surface of the cell achieved through the adhesins present on the bacterial surface^7^,^8^. From the many adhesins present in *H. pylori*, BabA, SabA, AlpA/B, and OipA are those studied most. They recognize special carbohydrates on the surface of epithelial cells, such as Lewis b (BabA) or sialyl-Lewis x (SabA)^9^. Recently also the HopQ adhesin has been added to the list and shown to support the injection of CagA^10^.

The CagA protein is the 4^th^ most abundant protein produced by *H. pylori* strain 26695, ranging behind GroEL (Hp0010), UreB (Hp0072) and TsaA (Hp1563)^11^. Since CagA is the only effector protein known being injected by the *cag*-T4SS, these very high quantities of CagA protein produced by *H. pylori* imply that a high amount of CagA can be injected into eukaryotic cells in order to see the effects described above. Having this in mind, we wanted to determine how much CagA is really injected during *in vitro* infection of *H. pylori* in comparison to the total amount of CagA produced. However, a quantitative approach to determine how much CagA is injected into the host cell has not been described. This is in contrast to *Salmonella* species using a Type III secretion system for effector protein injection^12^,^13^. In some of these systems the removal of adherent bacteria was achieved by different washing steps, which is not possible for *H. pylori* because of its tight binding to AGS cells. We therefore developed a novel procedure, which makes use of Proteinase K digestion to remove all bacteria from the surface of the cells without damaging the integrity of the eukaryotic cell. Using this technique and semi-quantitative analysis of western blots we have determined the amounts of CagA injected in the host cells during infection of gastric epithelial AGS cells *in vitro*.

## Results

### Proteinase K treatment removes bound bacteria from infected AGS cells

**Figure 1:**
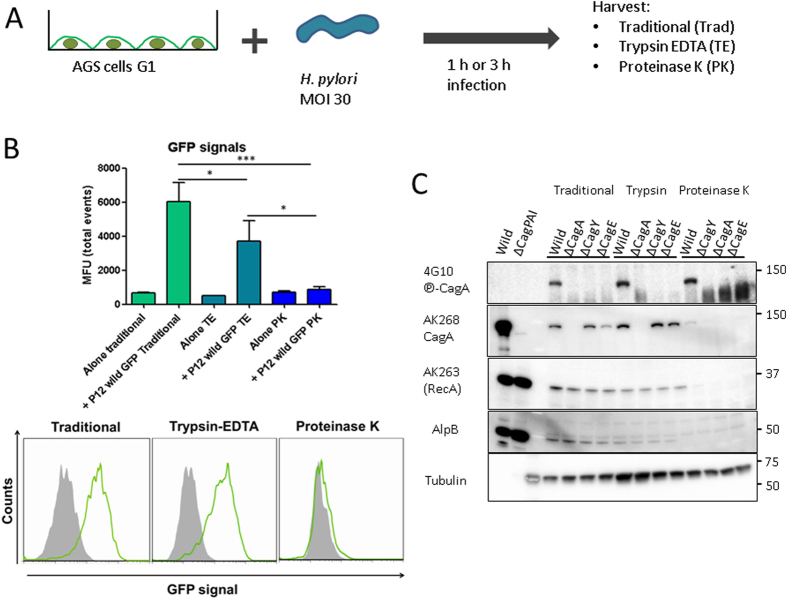
Establishment of different harvesting methods for the quantification of CagA injection. (**A**) Schematic representation of the experimental setup explained in Material and Methods section. (**B**) GFP expressing P12 strain (P12 GFP) infected AGS cells for one hour. Cells were harvested by scrapping (Traditional, Trad), Trypsin-EDTA (TE) or Proteinase K (PK) digestion. Mean fluorescence Units are shown here. Cells without infection are shown as control for changes in fluorescence caused by the different treatments. n = 3, ANOVA used for the statistical analysis. *P < 0.05, ***P < 0.001. Histograms are representative of data obtained. (**C**) Immunodetection of bacterial proteins phosphorylated CagA (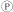-CagA, 4G10), CagA (AK268), RecA (AK263), AlpB, and cellular protein Tubulin. All detections shown here were done from the same blot transfer. Bacteria lysates from strain 26695 (and ΔCagPAI) were used as control for the western blots.

*H. pylori* shows a tight binding to host cells, which is mediated by a variable set of highly efficient bacterial adhesins^14^. This tight interaction of the bacteria with the cells does not allow removal of bacteria after infection by conventional physical methods without damaging the host cells. For this we developed a Proteinase K digestion protocol that achieves removal of bacteria without causing damage to the integrity of the membrane of the eukaryotic cell. The integrity of the eukaryotic cells was tested using propidium iodine (data not shown). We used serum-starved gastric adenocarcinoma epithelial cells (AGS) after 30 min of serum addition, at which point a high amount of ITGB1 was present on the surface of the cells. The infection proceeded for 1 or 3 hours with a multiplicity of infection (MOI) of 30. This was the lowest MOI to be used in a one hour infection experiment of synchronized cells allowing the immunodetection of the phosphorylated form of CagA. Higher MOIs did not allow a complete removal of *H. pylori* from AGS cells without causing severe damage of the host cell membrane by extended digestion times. Cells were then harvested by scrapping (Traditional, Trad), by Trypsin-EDTA detachment (TE/ Trypsin) or by Proteinase K digestion (PK) in 16 °C, to block uptake of bacterial fragments by the cell via endocytosis (Fig. 1A). To verify the removal of bacteria, we infected cells with an *H. pylori* strain expressing GFP in the cytoplasm (strain P12 GFP)^7^,^15^. After one hour of infection, cells were harvested, washed and the amount of bound bacteria was analyzed by flow cytometry. As shown in Fig. 1B, the proteinase K treatment removed all GFP signals, while TE treatment reduced about 40% of the signals in comparison to the traditional harvest, which represents all bacteria that remain bound to cells after the infection time and subsequent washing steps.

To extend the procedure to another *H. pylori* (strain 26695) and to verify that all bacterial proteins on the cell surface were removed as well, infected AGS cells were harvested using the different procedures described, and the lysates were analyzed by western blot for the presence of bacterial proteins RecA (cytoplasmic), AlpB (outer membrane) and CagA (cytoplasmic / outer membrane). RecA and AlpB were absent from the samples treated with Proteinase K only and the AlpB signal was strongly reduced by Trypsin-EDTA treatment. The tubulin signal shows that intracellular host cell proteins were conserved during the different treatments and harvest methods (Fig. 1C).

### Bacterial CagA is removed, but injected phosphorylated CagA is conserved after proteinase K treatment

**Figure 2:**
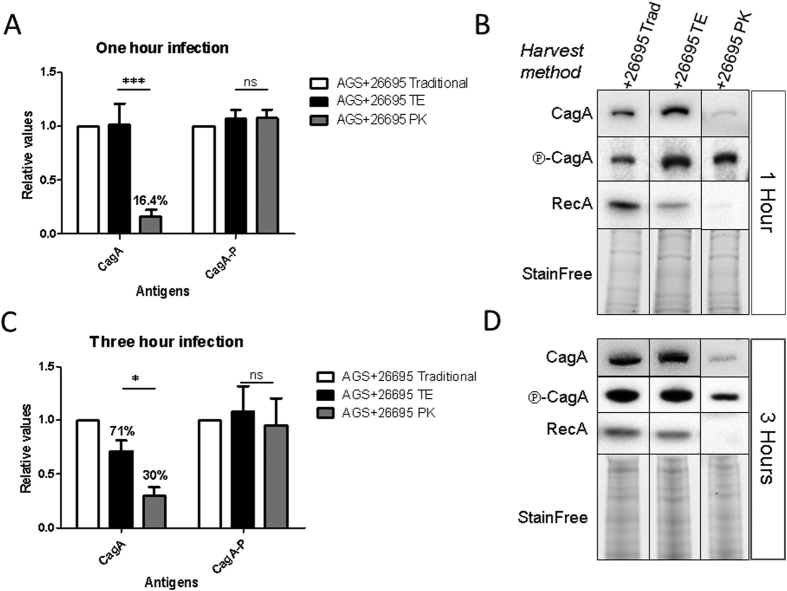
Quantification of signals for phosphorylated CagA (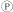-CagA) and CagA. Infection times were chosen for 1 hour (**A**,**B**) or 3 hours (**C**,**D**). Densitometry analysis of the signals from the immunodetection of CagA and its phosphorylated form 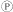-CagA. Signals from traditional harvest by scrapping (Trad) were used as normalization parameter stabilizing the 100% of the signals to be obtained. Signals obtained from samples digested Trypsin EDTA and Proteinase K are shown relative to the traditional harvest. Single examples of the blots for each time point are shown as well (**B**,**D**). All densitometry values were normalized to the Stain Free signal.

After one hour of infection, the signals for the phosphorylated form of CagA (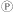-CagA) stayed rather constant in all treatment/harvest methods. However the signals detecting the CagA protein (CagA) are strongly reduced after proteinase K treatment (Fig. 1C). Quantification of the corresponding bands in the western blot (Fig. 2A) from three different experiments normalized to the signals of the traditional harvest after one hour infection clearly demonstrates that the procedure efficiently removed CagA in the bacteria bound to the cells. However the injected and phosphorylated CagA was completely protected, as no changes on CagA-phosphorylation signals were observed (Fig. 2B).

We next determined the amount of CagA after 3 hours of infection, since at this time point the effects on cytoskeletal changes caused by CagA translocation (e.g. hummingbird phenotype) are visible. Notably, the same conservation of CagA phosphorylation was observed with the different harvest treatments at the 3 hours time point (Fig. 2C). In contrast to the 1 hour infection, the levels of total CagA protein present in the lysates after Trypsin EDTA treatment (protease control treatment) of the 3 hour infections reached only 71% of the signal achieved with the traditional harvest.

In a further step we quantified translocated (phosphorylated CagA protein signal (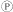-CagA)) versus total available/detected CagA (CagA protein signal (CagA)) and normalized it to the traditional harvest signals (which represent the bacteria adherent to the AGS cells, and all CagA available during the infection). After one hour of infection the CagA signal present after the total removal of bacteria, meaning that it is present inside the AGS cells, corresponded to approx. 16% of the total CagA signal observed in the traditional or TE treatments. As expected, after 3 hours of infection there was a higher amount of CagA protein inside the cell, corresponding to roughly 30% of the total CagA signal of the traditional harvest (Fig. 2C). Furthermore, after 3 hours of infection the CagA signal during TE treatment diminished (Fig. 2C), in contrast to the analysis of the signals of one hour infections, which did not show visible differences between Traditional harvest and TE. We hypothesize that during the three hours of infection, more CagA will be transported onto the surface of the bacteria and in this way it will be susceptible for digestion by trypsin. We tried to verify this by immunodetection of CagA comparing the one hour vs. the three-hour infection signals, but the Trypsin EDTA treatment of infected cells caused an unspecific binding of the antibodies to eukaryotic cells making it impossible for detection (data not shown). Remarkably, regardless of the time point or the kind of harvest, there was no difference in the signals detecting the phosphorylated CagA protein, which corroborated the comparability and reproducibility of the method used.

### Only a low percentage of *H. pylori* bacteria are ready to interact with ITGB1

**Figure 3:**
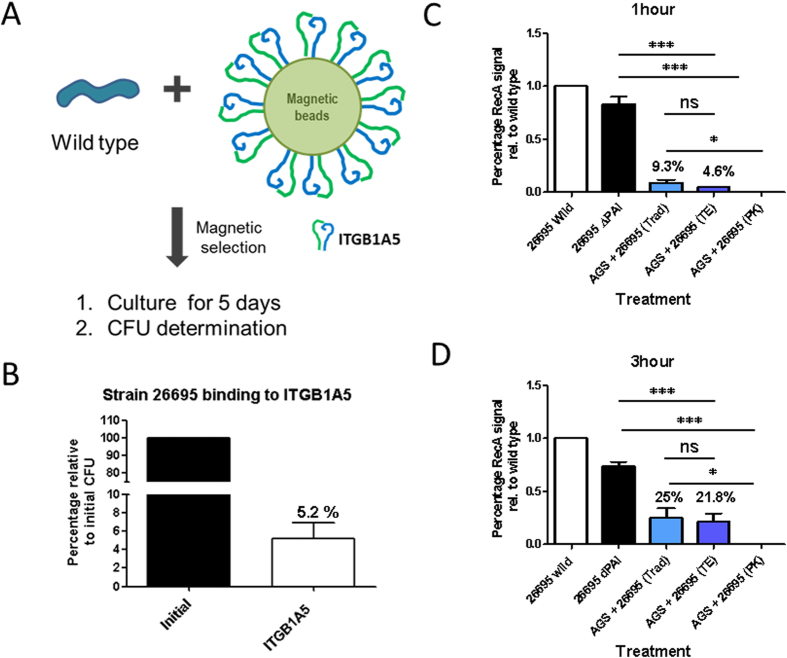
Availability of Cag apparatus and binding capacity of *H. pylori* in infection assays. (**A**) Scheme for the binding test of strain 26695 to ITGB1A5 coated beads (See Material and Methods). (**B**) Percentage of colony forming units recovered after binding assay to ITGB1A5 coated magnetic beads, relative to the total amount of bacteria present in the initial suspension. n = 4. T-test was used for the statistical analysis. (**C**,**D**) Quantification of RecA signals by densitometry analysis and after normalization to stain free signals, the percentage were calculated relative to the signals given by the lysates of the original amount of bacteria. Statistical analysis applied was one way ANOVA, with post-Turkey test for comparison between each different treatment. n = 3. *P < 0.05; **P < 0.01; ***P < 0.001, ns (not significant) P > 0.05.

The *cag*-T4SS injects CagA in the host cell after interaction with ITGB1^7^,^8^. Based on the data above, the low amount of injected CagA will indicate a low CagA translocation capacity or a low number of Cag T4SS apparatus able to inject the toxin. To determine the amount of bacteria grown on plates ready to interact with the T4SS receptor ITGB1, we looked at the amount of bacteria interacting with Integrin beta 1 alpha 5 (ITGB1A5)-coated magnetic beads (scheme in Fig. 3A). Based on our previously published AGS cell co-infection experiments with different *H. pylori* strains and their consequences for CagA translocation^15^, we assume that the interaction of the assembled Cag apparatus with the ITGB1 has to be a fast process. Therefore the interaction was tested using a window of 5 min for binding. Our results show that approx. 5% of the bacteria growing on media free of serum proteins show the ability to interact directly with the ITGB1 beads (Fig. 3B) in these 5 minutes of contact. We conclude that under these *in vitro* growth conditions of *H. pylori* 26695 only 5% of the bacteria have the Cag apparatus fully assembled and ready for integrin interaction.

### A low percentage of *H. pylori* is able to bind tightly to AGS cells after 1 and 3 hour infections

The translocation of CagA requires not only a functional secretion system, but equally important requires a certain capacity of adhesion of the bacteria to the host cell, as it has been shown for certain outer membrane proteins influencing the capacity of CagA translocation^10^,^16^. Therefore we determined the amount of bacteria bound to the cells by quantitative analysis of the RecA signals compared to the total RecA signals of the bacteria added at the moment of infection. The RecA signal of the bacteria bound to the cells harvested by scrapping (Traditional harvest, Trad) after one hour represents only 9.3% of the total signal obtained by all bacteria used for infection (Fig. 3C). Although the Trypsin EDTA treatment seems to leave only 4.6% of the signal, it is not statistically significant to the traditional harvest signal. As expected after Proteinase K treatment, no quantifiable signal could be detected. In the case of three hour infection (Fig. 3D), there are equally higher signals of RecA for both harvest systems, the Traditional and Trypsin-EDTA. We can conclude from these data that the longer the *in vitro* infection takes place, the better will be the binding of the bacteria to the cells and more CagA is translocated, explaining the rise in translocated CagA from 16% to 30%. However it is important to highlight that only a small fraction of the total amount of bacteria added to the infection assay are able to bind to cells during the two infection times tested.

## Discussion

Over the years many functions have been assigned to the CagA toxin of *Helicobacter pylori.* Among others it confers advantage to bacteria during infection of host tissue in iron-deficient conditions^17^, it causes changes on the chemokine profile of tissue increasing inflammation^14^, it induces alteration of the immune system by affecting the survival of B cells^18^ and changes the histological characteristics of the stomach^19^. All these effects of CagA are thought to finally lead to the formation of gastric cancer. It is therefore necessary to know the real amount of CagA entering the cells to evaluate its effectiveness. By removing the bacteria from the surface, we can not only evaluate the amount of CagA injected into the host cell, but we also can infer on the efficiency of the injected toxin by analyzing the amount of bacteria that remain tightly attached to the AGS cells. Since *H. pylori* needs to attach to the cells in order to inject the CagA, we used the analysis of the RecA protein signal to determine the percentage of bacteria tightly bound to the cells after the different infection times. From the initial amount of bacteria added, about 4–9% bound tightly to the cells after 1 hour of infection and 25% after 3 hours. At the same time, the amount of CagA injected after one hour was around 16% of the total CagA present in the adherent bacteria, and 30% after three hours. Considering total amount of bacteria in the infection and the amount of CagA injected by the bound bacteria we can assume that after one hour only 1.5% of the total CagA present in all bacteria during the *in vitro* infection is injected into the host cell and after 3 hours, 7.5% of the total CagA has been injected. The relatively low amount of injected versus total CagA, especially in the 1 hour infection experiments, may be explained as the product of a low number of bacteria having the Cag T4SS functionally assembled on their surface (~5%) and the low number of adherent bacteria (~4–9%). These values demonstrate how successful the toxin can be in modifying the cultured cells despite its small amount. Additionally, although we cannot define a kinetic of injection with two time points, it is interesting to observe that the more the bacteria are able to bind to the cells, the higher is the amount of CagA present inside them, confirming the necessity of binding for CagA translocation.

An important question arising is how do the *in vitro* CagA translocation results relate to the *in vivo* behavior of the bacteria? So far no reliable method has been described showing CagA translocation and its quantification under *in vivo* conditions in the infected host or experimental animal. We therefore cannot mirror any *in vitro* CagA translocation data with the situation *in vivo*. However the molecular mechanisms involved can be better understood in cell culture experiments and they have been used as a valid approximation to the situation *in vivo*. Also the question whether all CagA translocated into the host cell will be phosphorylated by the host cell kinases has to be left open. Each status of CagA might have its function, especially since phosphorylated and non-phosphorylated CagA (e.g. the latter with non-phosphorylatable EPIFA motif) have been reported to affect different cellular processes once inside the cell^4^,^20^,^21^. Furthermore, we cannot exclude that these large quantities of CagA toxin might have other functions besides its role in the host cell cytoplasm. Since CagA has been considered to be toxic only after injection into the cytoplasm of the host cell by a functional T4SS, the question arises what happens to the non-injected CagA? Does it stay in the bacteria? We know that small amounts are located on the bacterial surface. Can it also be secreted into the local “environment” during an infection? Would it be taken up by immune cells? Another aspect to be considered is a kind of regulation of CagA injection. We recently have discovered a bacteria-induced resistance mechanism of the cell to CagA injection^15^. Is the rather low amount of translocated CagA the result of a cellular “bottleneck” or a self-limitation of the bacteria to avoid a too strong damage of the host cell? Future studies should answer these and other unsolved questions around the role of CagA toxin in the relationship between host and *H. pylori.*

## Materials and Methods

### Bacteria strains and culture

The *H. pylori* strain P12 was isolated from a duodenal ulcer patient, and to produce a GFP protein it has been inserted a pHel4 plasmid containing the GFPmut3 protein under the AlpA promoter. The strain 26695 is a lab strain. The mutants 26695 Δ*hp0547*(*cagA*), Δ*hp0527*(*cagY*) and Δ*hp0544*(*cagE*) have been already described^22^. All *H. pylori* strains were grown on GC agar plates supplemented with a cholesterol/lipid mix (Gibco, Life Technologies) and vitamin mix as described^23^. All antibiotics were obtained from Sigma-Aldrich (Deisenhofen, Germany). Incubation of the bacteria was performed at 37 °C for 24 hours in an anaerobic incubator containing a controlled atmosphere composed of of 5% O_2_, 10% CO_2_ and 85% N_2_ (Oxoid, Wesel, Germany).

### Binding assays with Magnetic beads

Chemicell magnetic beads were coated with recombinant ITGB1A5^24^ following the 2-step protocol recommended by the manufacturer. As blocking buffer we used 25 mM Tris-HCl pH7.5 (BSA shows unspecific binding to *H. pylori*). Bacteria were suspended in PBS buffer (Ca-, Mg-) from Gibco and OD_550_ measured. Bacteria suspension of OD_550_ 0.1 were exposed to 10 μl of beads for 5 min and thoroughly washed three times. Serial dilutions were plated from the original OD_550_ 0.1 and the magnetic beads suspended in 1 ml. After plating of bacterial suspensions, they were incubated for 5 days at 10% CO_2_, 37 °C and colony forming units (CFUs) were quantified.

### Infections assay

All infections were done as described^15^. Shortly, we used AGS cells (ATCC CRL 1739a human gastric adenocarcinoma epithelial cell line) grown on 6-well plates in RPMI media for at least 36 hours in 5% CO_2_, 37 °C to a density of approx. 80% confluence, and incubated further in serum-free media for at least 8 hours. Before infection, cells were incubated for 30 min in serum containing RPMI media (Complete media, CM) and infected with an MOI of 30 for 1 or 3 hours at 37 °C in 5% CO_2_.

### Harvest methods

At the moment of harvest, all cells were placed on ice, and washed twice with 2 ml cold PBS (Ca-, Mg-; Gibco, Life Technologies). Three treatments were used for harvest: i) Traditional (Trad), in which the cells were placed 5 hours in 16 °C and collected using cell scrapers to remove them from the growth surface in 1 ml cold PBS containing 1 mM PMSF, 1 mM Sodium Ortho-vanadat, 1 μM Leupetin and 1 μM pepstatin. ii) Trypsin-EDTA (TE), in which cells were removed from the surface using 0.5 ml of Trypsin-EDTA solution (Gibco, Life Technologies) and incubated for 5 hours at 16 °C, at which time they had lost adherence to the growth surface and where collected. iii) Proteinase K digestion, which it was achieved using 1 ml per well of a 10 mg/ml crude Proteinase K (Sigma) in Proteinase K buffer (50 mM Tris-HCl pH 7.5, 150 mM NaCl, 1 mM CaCl_2_, 1 mM MgCl_2_, 1 mM KCl) for 5 hours at 16 °C, after which cells were detached from the growth surface.

All detached cells through protease treatment, were collected and mixed 1:1 with a cold PBS solution containing 2 mM PMSF to inactivate the activity of the proteases. After all samples were collected, they were centrifuged once at 200 g 4 °C for 10 min in a swing rotor centrifuge. Pellets were washed 5 times using 1 ml cold PBS 1 mM PMSF with centrifugation steps of 10 min 100 g at 4 °C in a swing rotor centrifuge. Pellets were finally resuspended in 20 μl of PBS containing proteinase inhibitors and mixed with 25 μl of 2X SDS loading buffer. Samples were boiled for 10 min at 95 °C and store at −20 °C until needed.

### Flow cytometry

Infections and harvest were done as explained previously. For the conservation and stabilization of samples, after the last washing step, pellets were suspended in PBS containing 1% PFA solution and analyzed using a BD Canto II flow cytometer. For the statistical analysis, mean fluorescent units from each treatment were normalized to their controls (uninfected cells) and analyzed using an ANOVA one way analysis. The histograms are representative of data obtained in the experiments.

### Western blots and quantification of signals

Proteins were separated by 6% and 7% SDS-PAGES (Single gel system by Ahn T^25^ and blotted on PVDF membranes (Bio-Rad). CagA was detected with rabbit polyclonal antibodies against the N- or the C-terminal part and phosphorylated CagA and mouse monoclonal α-phosphotyrosine antibody 4G10 (Millipore/Upstate). RecA was detected using the antibody AK263^26^, AlpB using the antibody AK227^27^. Secondary antibodies used were horseradish peroxidase conjugated. For antibody detection Millipore Immobilon Western (Millipore) and GelDoc imaging (BioRad) were used. ImageLab software 4.1 (BioRad) and 5.1 versions were used for semi-quantitative analysis of chemoluminescent signals, using StainFree protein staining^28^ as normalization factor, with a background correction of 10.

For an accurate comparison of signals, all proteins were detected on the same membrane. The protocols and buffers here mentioned were taken and adapted from recommended protocol published by Millipore in its “Protein Blotting Handbook” (found online at http://www.merckmillipore.com/DE/en/life-science-research/protein-detection-quantification/western-blotting/protocols/q9ib.qB.710AAAFBRP0RRkww,nav). For reprobing of membranes without loss of signals, PVDF membranes were left to dry for 1 hour at 37 °C after blot transfer and reactivated with methanol for 15–30 s before starting detection. Between protein detections we achieved removal of previous primary antibodies using a solution containing 1% SDS, 25 mM Glycine, pH 2 for 1 hour at room temperature. The order of detection was: 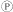-CagA (120–130 kDa, 4G10 (Millipore), mouse IgG), RecA (32 kDa, AK263, rabbit IgG), CagA (120–130 kDa, AK257, rabbit IgG), AlpB (51 kDa, AK277, Rabbit IgG) and Tubulin (68 kDa, (Sigma), mouse IgG).

## Additional Information

**How to cite this article**: Jiménez-Soto, L. F. and Haas, R. The CagA toxin of *Helicobacter pylori*: abundant production but relatively low amount translocated. *Sci. Rep.* **6**, 23227; doi: 10.1038/srep23227 (2016).

## References

[b1] Odenbreit S. *et al.* Translocation of Helicobacter pylori CagA into gastric epithelial cells by type IV secretion. Science 287, 1497–1500 (2000). 10688800 10.1126/science.287.5457.1497

[b2] Selbach M., Moese S., Hauck C. R., Meyer T. F. & Backert S. Src is the kinase of the Helicobacter pylori CagA protein *in vitro* and *in vivo*. J Biol.Chem. 277, 6775–6778 (2002). 11788577 10.1074/jbc.C100754200

[b3] Poppe M., Feller S. M., Romer G. & Wessler S. Phosphorylation of Helicobacter pylori CagA by c-Abl leads to cell motility. Oncogene. 26, 3462–3472 (2007). 17160020 10.1038/sj.onc.1210139

[b4] Bagnoli F., Buti L., Tompkins L., Covacci A. & Amieva M. R. Helicobacter pylori CagA induces a transition from polarized to invasive phenotypes in MDCK cells. Proc.Natl.Acad.Sci.USA 102, 16339–16344 (2005). 16258069 10.1073/pnas.0502598102 PMC1274241

[b5] Saadat I. *et al.* Helicobacter pylori CagA targets PAR1/MARK kinase to disrupt epithelial cell polarity. Nature 447, 330–333, 10.1038/nature05765 (2007). 17507984

[b6] Backert S., Tegtmeyer N. & Selbach M. The versatility of Helicobacter pylori CagA effector protein functions: The master key hypothesis. Helicobacter 15, 163–176, 10.1111/j.1523-5378.2010.00759.x (2010). 20557357

[b7] Jimenez-Soto L. F. *et al.* Helicobacter pylori type IV secretion apparatus exploits beta1 integrin in a novel RGD-independent manner. PLoS Pathog 5, e1000684, 10.1371/journal.ppat.1000684 (2009). 19997503 PMC2779590

[b8] Kwok T. *et al.* Helicobacter exploits integrin for type IV secretion and kinase activation. Nature. 449, 862–866 (2007). 17943123 10.1038/nature06187

[b9] Backert S., Clyne M. & Tegtmeyer N. Molecular mechanisms of gastric epithelial cell adhesion and injection of CagA by Helicobacter pylori. Cell communication and signaling: CCS 9, 28, 10.1186/1478-811X-9-28 (2011). 22044679 PMC3266215

[b10] Belogolova E. *et al.* Helicobacter pylori outer membrane protein HopQ identified as a novel T4SS-associated virulence factor. Cell Microbiol, 10.1111/cmi.12158 (2013). PMC3797234 23782461

[b11] Jungblut P. R. *et al.* Comparative proteome analysis of Helicobacter pylori. Mol Microbiol 36, 710–725 (2000). 10844659 10.1046/j.1365-2958.2000.01896.x

[b12] Schlumberger M. C. *et al.* Real-time imaging of type III secretion: Salmonella SipA injection into host cells. Proc Natl Acad Sci USA 102, 12548–12553, 10.1073/pnas.0503407102 (2005). 16107539 PMC1194920

[b13] Van Engelenburg S. B. & Palmer A. E. Quantification of real-time Salmonella effector type III secretion kinetics reveals differential secretion rates for SopE2 and SptP. Chem Biol 15, 619–628, 10.1016/j.chembiol.2008.04.014 (2008). 18559272 PMC3297674

[b14] Posselt G., Backert S. & Wessler S. The functional interplay of Helicobacter pylori factors with gastric epithelial cells induces a multi-step process in pathogenesis. Cell communication and signaling: CCS 11, 77, 10.1186/1478-811X-11-77 (2013). 24099599 PMC3851490

[b15] Jimenez-Soto L. F., Clausen S., Sprenger A., Ertl C. & Haas R. Dynamics of the Cag-type IV secretion system of Helicobacter pylori as studied by bacterial co-infections. Cell Microbiol, 10.1111/cmi.12166 (2013). 23844976

[b16] Ishijima N. *et al.* BabA-mediated adherence is a potentiator of the Helicobacter pylori type IV secretion system activity. J Biol Chem 286, 25256–25264, 10.1074/jbc.M111.233601 (2011). 21596743 PMC3137096

[b17] Tan S., Noto J. M., Romero-Gallo J., Peek R. M. Jr. & Amieva M. R. Helicobacter pylori perturbs iron trafficking in the epithelium to grow on the cell surface. PLoS Pathog 7, e1002050, 10.1371/journal.ppat.1002050 (2011). 21589900 PMC3093365

[b18] Umehara S., Higashi H., Ohnishi N., Asaka M. & Hatakeyama M. Effects of Helicobacter pylori CagA protein on the growth and survival of B lymphocytes, the origin of MALT lymphoma. Oncogene 22, 8337–8342 (2003). 14614457 10.1038/sj.onc.1207028

[b19] Murata-Kamiya N. *et al.* Helicobacter pylori CagA interacts with E-cadherin and deregulates the beta-catenin signal that promotes intestinal transdifferentiation in gastric epithelial cells. Oncogene. 26, 4617–4626 (2007). 17237808 10.1038/sj.onc.1210251

[b20] El-Etr S. H., Mueller A., Tompkins L. S., Falkow S. & Merrell D. S. Phosphorylation-Independent Effects of CagA during Interaction between Helicobacter pylori and T84 Polarized Monolayers. Journal of Infectious Diseases 190, 1516–1523, 10.1086/424526 (2004). 15378446

[b21] Amieva M. R. *et al.* Disruption of the epithelial apical-junctional complex by Helicobacter pylori CagA. Science 300, 1430–1434, 10.1126/science.1081919 (2003). 12775840 PMC3369828

[b22] Fischer W. *et al.* Systematic mutagenesis of the Helicobacter pylori cag pathogenicity island: essential genes for CagA translocation in host cells and induction of interleukin-8. Mol.Microbiol. 42, 1337–1348 (2001). 11886563 10.1046/j.1365-2958.2001.02714.x

[b23] Jimenez-Soto L. F. *et al.* Effects of cholesterol on Helicobacter pylori growth and virulence properties *in vitro*. Helicobacter 17, 133–139, 10.1111/j.1523-5378.2011.00926.x (2012). 22404444

[b24] Takagi J., Erickson H. P. & Springer T. A. C-terminal opening mimics ‘inside-out’ activation of integrin alpha5beta1. Nat Struct Biol 8, 412–416, 10.1038/87569 (2001). 11323715

[b25] Ahn T., Yim S. K., Choi H. I. & Yun C. H. Polyacrylamide gel electrophoresis without a stacking gel: use of amino acids as electrolytes. Anal Biochem 291, 300–303, 10.1006/abio.2001.5038 (2001). 11401306

[b26] Fischer W. & Haas R. The RecA protein of Helicobacter pylori requires a posttranslational modification for full activity. J.Bacteriol. 186, 777–784 (2004). 14729704 10.1128/JB.186.3.777-784.2004 PMC321478

[b27] Odenbreit S., Till M., Hofreuter D., Faller G. & Haas R. Genetic and functional characterization of the alpAB gene locus essential for the adhesion of Helicobacter pylori to human gastric tissue. Mol.Microbiol. 31, 1537–1548 (1999). 10200971 10.1046/j.1365-2958.1999.01300.x

[b28] Ladner C. L., Yang J., Turner R. J. & Edwards R. A. Visible fluorescent detection of proteins in polyacrylamide gels without staining. Anal Biochem 326, 13–20, 10.1016/j.ab.2003.10.047 (2004). 14769330

